# Pathophysiologic Characterization of a Novel Rabbit Model of Biliary Tract Infection-Derived Sepsis

**DOI:** 10.1038/s41598-019-48462-0

**Published:** 2019-08-16

**Authors:** Liangshuo Hu, Yichao Chai, Rui Xi, Haoyang Zhu, Yue Wang, Fenggang Ren, Jing Zhang, Zhao Xue, Hongke Zhang, Rongqian Wu, Yi Lv

**Affiliations:** 1 Department of Hepatobiliary Surgery, First Affiliated Hospital, Xi’an Jiaotong University, Xi’an, 710061 Shaanxi China; 2 National Local Joint Engineering Research Center for Precision Surgery & Regenerative Medicine, First Affiliated Hospital, Xi’an Jiaotong University, Xi’an, 710061 Shaanxi China; 3 Shaanxi Provincial Center for Regenerative Medicine and Surgical Engineering, First Affiliated Hospital, Xi’an Jiaotong University, Xi’an, 710061 Shaanxi China; 4 Department of Hepatobiliary Surgery, Central Hospital of Hanzhong, Hanzhong, 723000 Shaanxi China; 5 Department of Thyroid Breast Surgery, West China Hospital of Sichuan University, Chengdu, 610041 Sichuan China; 6 Department of Oncology Surgery, Second Affiliated Hospital, Xi’an Jiaotong University, Xi’an, 710004 Shaanxi China

**Keywords:** Metabolism, Gastrointestinal system

## Abstract

Biliary tract infection (BTI)-derived sepsis remains a serious problem with significant morbidity and mortality in the modern era of critical care management. Current animal models of BTI have relied mostly on injecting purified bacteria or their toxins into the biliary tract. These models do not fully reflect pathophysiology or disease processes of clinical cholangitis or cholecystitis. In the current study, we developed a novel model of BTI by performing cholecystocolonic anastomosis (CCA) in rabbits and characterized pathophysiologic changes in this model. This model is intended to mimic the clinical process of cholecystocolonic fistula with reflux cholangitis, a severe form of BTI. Adult male rabbits were subjected to BTI-derived sepsis through an anastomosis of the gall bladder to the colon (i.e., CCA). The animals were monitored for 7 days to record survival. In additional groups of animals, various bacterial, hemodynamic, histological and biochemical parameters were measured at 12, 24, 48 and 72 h after CCA. The anastomosis between the gallbladder and the colon required about 5–8 min to finish. The median survival time for rabbits after CCA was 96 h. The positive rates of bacterial culture at 72 h after CCA were 83.3% and 100% in the blood and liver, respectively. The most common microorganism was *Escherichia coli* followed by *Enterococcus*. Plasma Tumor Necrosis Factor-α (TNF-α), Lnterleukin-10 (IL-10), Lnterleukin-6 (IL-6), and High-mobility group box 1 protein (HMGB-1) levels were greatly elevated after CCA. The cardiac index and heart rate increased slightly at 12 h after CCA and then continued to decrease. Systemic hypotension developed 48 h after CCA. Histological studies showed reflux cholangitis with acute lung and kidney injury. Cholecystocolonic anastomosis produces polymicrobial sepsis in rabbits, which mimics many aspects of human BTI-derived sepsis. It is reproducible and easy to perform and may serve as an excellent model for future sepsis research.

## Introduction

Biliary tract infection (BTI), including cholangitis and cholecystitis, is a common and serious condition. In severe cases, sepsis and septic shock could be triggered if infection breaks the hepatic immunity barrier. Significant proportion of BTI patients develops bacteremia and subsequent sepsis^1–3^. It is the second most common cause of sepsis in the elderly population^4,5^. Patients with BTI-derived sepsis are associated with high mortality and remain a substantial therapeutic challenge^6,7^. As such, there is an urgent need to understand the pathophysiologic changes in BTI-derived sepsis.

The purpose of using a reproducible animal model is to have a controlled setting that decreases the number of variables so that one can study in detail the mechanisms responsible for the altered immunological, cardiovascular, and metabolic changes under those conditions and devises better and more effective therapeutic modalities. Current animal models of BTI have relied mostly on injecting purified bacteria or their toxins into the biliary tract^8,9^. These models have provided valuable information regarding the mechanisms responsible for cell and organ dysfunction under such conditions. However, human cases of BTI are usually caused by a nidus of infection with replicating bacteria that persists for an extended time. Therefore, these models do not fully reflect pathophysiology or disease processes of clinical cholangitis or cholecystitis.

Rodents are the most widely used animals for medical research. As low-level mammals, however, their pathophysiologic responses are markedly different from those of humans. The debate about how well rodent models mimic human inflammatory diseases is still ongoing^10,11^. Many researchers tried to avoid this problem by using dog, pig, and even horse models. However, these animals need special experimental conditions and are usually associated with high costs, which significantly limit the use of these models. Rabbits are medium-sized mammals and very economical compared with the expense of larger animals. Due to their similar physiology to that of humans, rabbits have been widely used in research on hepatic and biliary disease^12,13^.

Microbiological studies indicated that fecal microbiota appears to be the major source of biliary tract infections^14,15^. Enterogenous bacteria attack the liver though the biliary tract and lead to acute liver injury. In the current study, we developed a novel model of BTI-derived sepsis by performing cholecystocolonic anastomosis (CCA) in rabbits and characterized pathophysiologic changes in this model. This model is intended to mimic the clinical process of cholecystocolonic fistula with reflux cholangitis, a severe form of BTI.

## Methods

### Experimental animals

All animal procedures were performed in accordance with the guidelines of the China Council on Animal Care and Use and approved by the Institutional Ethics Committee of the Xi’an Jiaotong University (IECXJTU), Shannxi, China (№. XJTULAC2017-725). A total of 46 adult male Japanese white rabbits with a mean weight of 2.5 kg served as subjects in this study. Rabbits were fed a standard laboratory diet with water and food *ad libitum* and were housed singly in standard cages under constant environmental conditions with 24 ± 2 °C, 50 ± 20% humidity and a 12-h light-dark cycle. Before surgery, 40 rabbits randomly were subjected to BTI models (12 h, 24 h, 48 h, and 72 h, 10 rabbits per group) and 6 for sham operation.

### Rabbit model of BTI

The rabbits were subjected to BTI by CCA. Briefly, rabbits were fasted for at least 4 hours prior to the induction of anesthesia. The anesthesia was induced by isoflurane (5%) inhalation and maintained by intravenous injection of ketamine (50 mg/kg) and medetomidine (2 mg/kg). The ventral neck, abdomen and groin were shaved and washed with 10% povidone iodine. A 6-cm midline abdominal incision was performed. A 16G catheter was inserted into the proximal common bile duct near the duodenum for intestinal content injection. The distal part of the bile duct was ligated with a 3-0 silk ligature. An anastomosis was created from the underside of the gallbladder to the colon at 10 cm proximal to the colon-cecal valve. The length of the anastomosis was 8–10 mm. Then, 1 g of intestinal content was diluted with 2 ml of sterile saline solution. After the mixture was allowed to stand for 10 min, 1 ml of the supernatant was gently injected into the bile duct system through the 16 G catheter placed earlier. Then the catheter was removed and the proximal common bile duct was closed with double ligation. Sham-operated animals (i.e., normal control groups) underwent the same surgical procedure with the exception that the anastomosis between the gallbladder and the colon was not performed and the intestinal content was not injected. All procedures were performed in strict accordance with the principles of antisepsis. The rabbits were observed during the anesthetic recovery until fully awaked and provided 24 hours of postoperative recovery time in a quiet, warm and dry area. Buprenorphine (0.03–0.05 mg/kg) was given subcutaneously every 12 h for post-operative analgesia. Six rabbits per group were subjected to hemodynamic measurement at various time points after the surgery. These animals were then euthanized with a lethal dose of sodium pentobarbital to harvest blood and tissue samples. The rest of the rabbits were allowed food and water *ad libitum* and were monitored for 7 d to record survival.

### Hemodynamic measurement

Mean arterial pressure and heart rate were measured with a catheter inserted into the femoral artery. Data were recorded onto the computer with an analog/digital transducer (MP100, TM-WAVE, China) and data processing software (BL420F, TM-WAVE, China). Resistance index (RI) of aorta and cardiac index were monitored by color Doppler ultrasound.

### Bacterial culture

Blood samples were delivered in culturing bottles and processed within 2 h of collection. Serial ten-fold dilutions of blood were made a series of 3 dilutions: 1:10, 1:100, and 1:000. Viable counts from these dilutions and the original specimens were set up on duplicate agar plates and incubated overnight at 37 °C. In addition, liver specimens were delivered in sterile containers. The tissue was weighed and crushed. Dilution and inoculation of the tissue homogenate was performed in the same manner as for blood specimens.

### Histological examination

Histological sections were reassessed by one board-certified pathologist who was blinded to the animal groups. To evaluate cholangitis in the liver, the grade of cholangitis was scored as described before^16,17^.

### Blood tests

Blood samples were harvested and plasma were immediately separated and stored at −80 °C until analysis. The complete blood count (CBC) test and biochemical analyses were monitored. Plasma Tumor Necrosis Factor-α (TNF-α), Lnterleukin-10 (IL-10), Lnterleukin-6 (IL-6), and High-mobility group box 1 protein (HMGB-1) concentrations were determined using a ELISA Kit.

### Statistical analysis

Statistical analysis was performed with SPSS 22.0 (SPSS, Chicago, IL, US) and GraphPad Prism 5.0 (GraphPad Software, San Diego, CA, US). Data are expressed as means ± SE. Statistical analysis was performed using Student’s t test for continuous variables in a paired and unpaired fashion, or analysis of variance (ANOVA) for multiple comparisons using Newman-Kuels test. Variables with a non-Gaussian distribution (Bacteria quantitation) were compared using one-way ANOVA nonparametric tests. All statistical tests were performed with 2-tailed distribution. Kaplan–Meier curves were created for survival and morbidity analysis in this study. *P* < 0.05 was considered statistically significant.

### Ethics approval and consent to participate

This study was reviewed and approved by the “Institutional Ethics Committee of the Xi’an Jiaotong University” (№. XJTULAC2017-725), and the project implementation process was in line with the ethical principles.

## Results

### CCA results in bacteremia and significant mortality in rabbits

**Figure 1:**
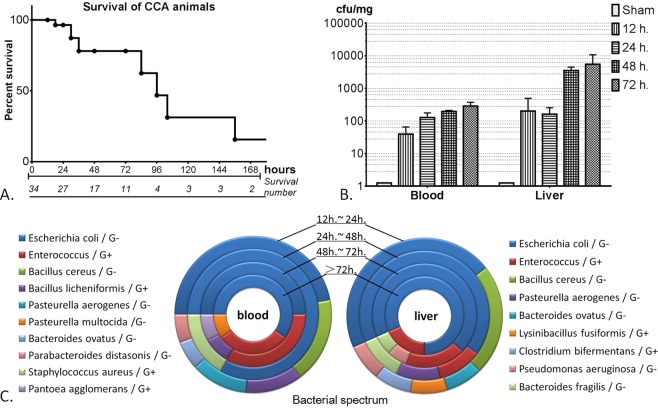
Survival curve and bacterial quantitation of the. (**A**) Survival curve for rabbits after modeling; (**B**) 36 rabbits (6 rabbits per group) were subjected to cholecystocolonic anastomosis and bacterial quantitation in blood and liver tissues were estimated. (**C**) Bacterial spectrum in blood and liver tissues after modeling.

The anastomosis between the gallbladder and the colon required about 5–8 min to finish. This model resulted in significant mortality in the rabbits (Fig. 1A). The overall 7-day survival rate was 15.6%. The median survival time after CCA was 96 h. Bacterial culture was performed at 12, 24, 48 and 72 h after CCA or sham operation. No bacteria were found in the blood and liver samples from sham-operated animals (Fig. 1B). The positive rates of bacterial culture at 72 h after CCA were 83.3% and 100% in the blood and liver, respectively. The bacterial counts appeared to increase along with time. The most common microorganism in the liver of CCA rabbits was *Escherichia coli* followed by *Enterococcus*. Other microbes found in the bacterial culture included *Bacteroides fragilis*, *Bacillus cereus*, and *Bacteroides ovatus*. Blood cultures share the same microorganisms as the liver, suggesting the bacteremia was BTI-derived.

### CCA initiates an inflammatory response in rabbits

**Figure 2:**
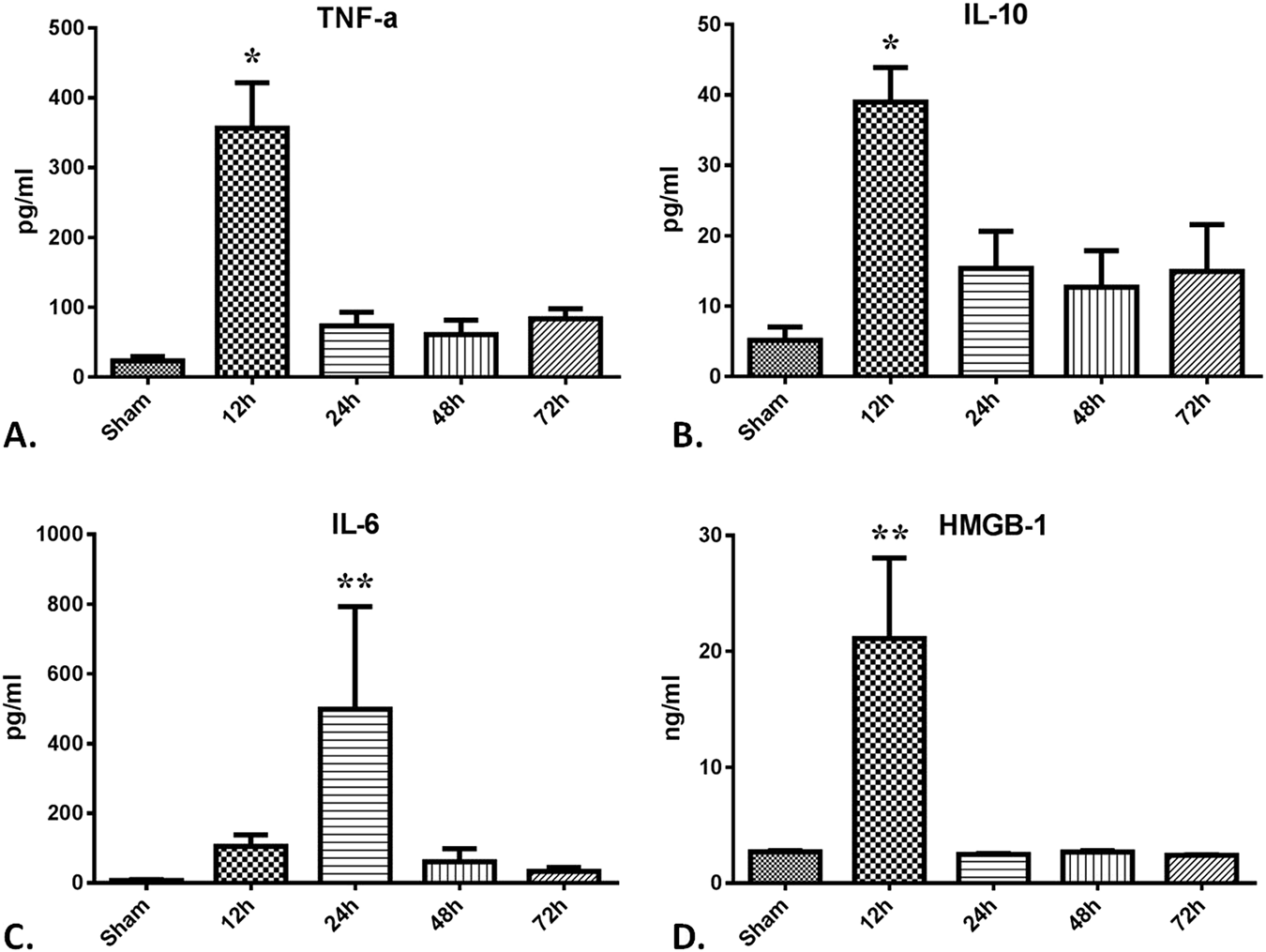
Increased expression of inflammatory mediators in the plasma after CCA modeling. 36 rabbits (6 rabbits per group) were subjected to cholecystocolonic anastomosis and plasma samples were harvested at each time point. TNF-α (**A**), IL-10 (B), IL-7 (**C**) and HMGB-1 (**D**) were measured as described in Materials and Methods. Data are presented as mean ± SD and compared using ordinary one-way analysis of variance and the Dunnett’s multiple comparisons test. P* < 0.05 vs group sham and group of the 12 h, 48 h, and 72 h time point. P** < 0.01 vs group sham and group of the 24 h, 48 h, and 72 h time point.

The complete blood count test showed that white blood cells appeared to increase along with time after CCA (*P* < 0.05). The number of erythrocytes and platelet along with the hemoglobin content appeared to decrease, while the number of neutrophils appeared to increase (Table 1). However, the changes did not reach statistically significant. As shown in Fig. 2A–D, concentrations of inflammatory mediators, including TNF-α, IL-10, IL-6, and HMGB-1, were found to be greatly elevated in the plasma after CCA (*P* < 0.001). Plasma concentrations of TNF-α, IL-10 and HMGB-1 were elevated but only at the 12 h after CCA (*P* < 0.01). Plasma levels of TNF-α rose from 23.3 ± 6.5 to 356.3 ± 65.1 pg/ml (Fig. 2A), IL-10 rose from 5.2 ± 1.9 to 39.0 ± 4.9 pg/ml and HMGB-1 rose from 2.7 ± 0.1 to 21.1 ± 6.9 ng/ml (Fig. 2B,D). IL-6 levels were found to have increased significantly, 7.2 ± 2.3 before surgery to 498.5 ± 294.3 pg/ml at 24 h after CCA (Fig. 2C, *P* < 0.05).

**Table 1:** Results of complete blood count test.

	Sham	12 hours	24 hours	48 hours	72 hours
Red blood cell count (10^12^/L)	5.63 ± 0.09	5.77 ± 0.27	5.95 ± 0.27	5.53 ± 0.31	4.89 ± 0.46
Hemoglobin (g/L)	115.6 ± 2.7	124.0 ± 7.0	117.0 ± 6.54	110.3 ± 5.6	99.0 ± 8.4
White blood cell count (10^9^/L)	6.56 ± 1.34	5.96 ± 2.17	8.28 ± 1.12	8.56 ± 1.53	9.95 ± 1.07*
Neutrophil granulocyte count (10^9^/L)	3.92 ± 1.03	3.96 ± 2.40	4.92 ± 0.98	5.16 ± 0.99	6.06 ± 0.93
Lymphocyte count (10^9^/L)	3.31 ± 0.65	3.47 ± 0.25	3.06 ± 0.43	2.44 ± 0.44	2.08 ± 0.26
Platelet count (10^9^/L)	276.5 ± 57.3	298.5 ± 137.5	260.8 ± 42.6	304.2 ± 70.9	160.0 ± 51.6

Values are presented as means ± SE. *Means P < 0.05.

### CCA produces an early hyperdynamic and late hypodynamic response in rabbits

**Figure 3:**
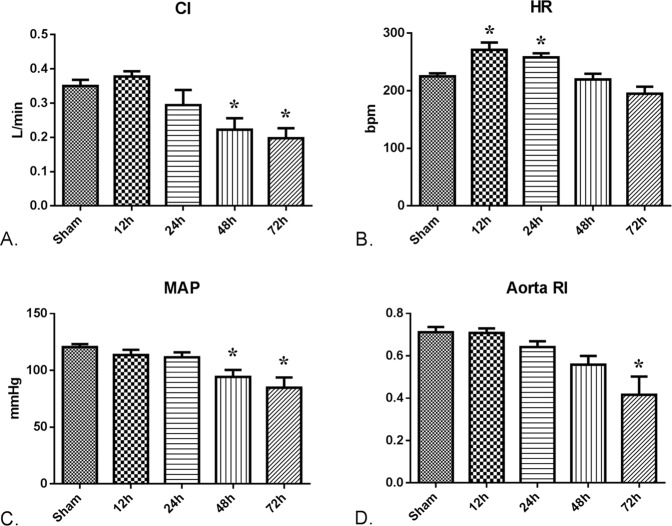
Hemodynamic responds after CCA modeling. 36 rabbits (6 rabbits per group) were subjected to cholecystocolonic anastomosis and hemodynamic tests were performed at each time point. Cardiac index (**A**), heart rate (**B**), mean arterial pressure (**C**) and aorta resistance index (**D**) were measured as described in Materials and Methods. Data are presented as mean ± SD and compared using ordinary one-way analysis of variance and the Dunnett’s multiple comparisons test. P* < 0.05 vs group of the 12 h time point, P** < 0.05 vs group at 24 h;

The cardiac index rose from 0.35 ± 0.02 to 0.38 ± 0.02 L/min at 12 h after CCA, but continued to decrease after that (Fig. 3A, *P* < 0.01). The heart rate was significantly elevated during the first 24 h after CCA and returned to the normal range at 48 and 72 h after CCA (Fig. 3B). The mean arterial pressure (MAP) did not significantly change during the first 24 h after CCA, but decreased considerably at 48 and 72 h after CCA (Fig. 3C, *P* < 0.001). Consistently, the aorta Resistance index (RI) also decreased gradually after CCA (Fig. 3D, *P* < 0.001).

### CCA leads to multiply organ injury in rabbits

**Figure 4:**
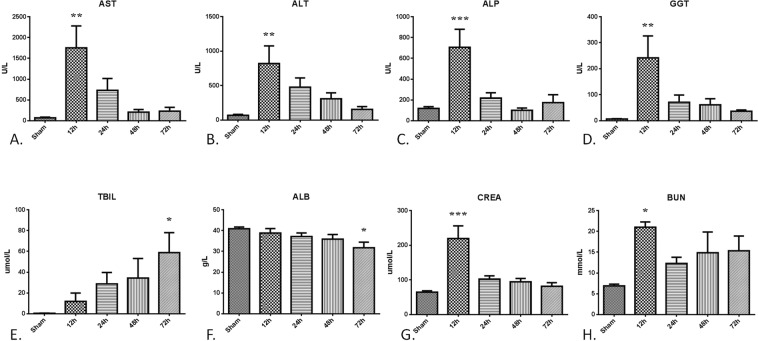
Liver and renal function tests. 36 rabbits (6 rabbits per group) were subjected to cholecystocolonic anastomosis and serum samples were harvested at each time point. Concentrations of aspartate aminotransferase (**A**), alanine amiotransferase (**B**), alkaline phosphatase (**C**), gamma-glutamyl transpeptidase (**D**), total bilirubin (**E**), albumin(**F**), creatinine (**G**) and blood urea nitrogen (**H**) in serum were measured. Data are presented as mean ± SD and compared using ordinary one-way analysis of variance and the Dunnett’s multiple comparisons test. P* < 0.05 vs group sham, P** < 0.05 vs group sham and group of the 48 h and 72 h time point, P*** < 0.05 vs group sham and group of the 24 h, 48 h, and 72 h time point;

As shown in Fig. 4A–D., the plasma levels of aspartate amino transferase (AST), alanine transaminase (ALT), alkaline phosphatase (ALP), and gamma-glutamyl transpeptidase (GGT) were found to be significantly increased at 12 h after CCA and then gradually decreased over time (All *P* < 0.001). Total bilirubin (TBIL) levels continued to increase after operation while albumin (ALB) levels showed the opposite trend (Fig. 4E,F, both *P* < 0.05). For the renal function test, significant increases in the plasma levels of creatinine (CREA) and blood urea nitrogen (BUN) were found at the 12 h post-CCA (Fig. 4G,H, both *P* < 0.001).

**Figure 5:**
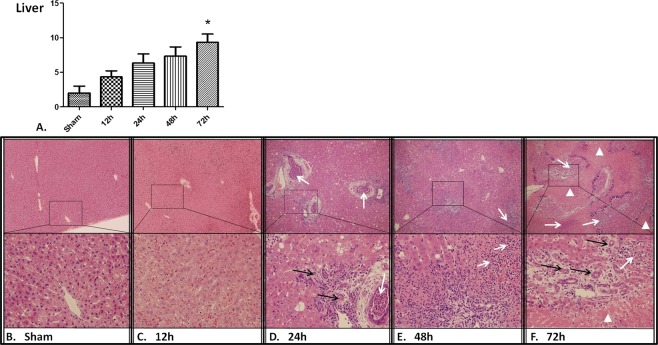
H&E staining of the liver tissue. 36 rabbits (6 rabbits per group) were subjected to cholecystocolonic anastomosis and liver tissue samples were harvested. The grade of cholangitis was found to be significantly increased over time (**A**). Data are presented as mean ± SD and compared using ordinary one-way analysis of variance and the Dunnett’s multiple comparisons test. P* < 0.05 vs group sham and group of the 12 h time point. H&E staining of the liver tissue at 12 h (**C**), 24 h (**D**), 48 h (**E**), and 72 h (**F**) time point after modeling versus sham group (**B**). The white arrows indicate a portion of inflammatory infiltration or purulence, the black arrows indicate hyperplasia of small bile ducts, and the white triangles indicate zone of extensive coagulative necrosis. (Original magnification: × 100 and × 400).

**Figure 6:**
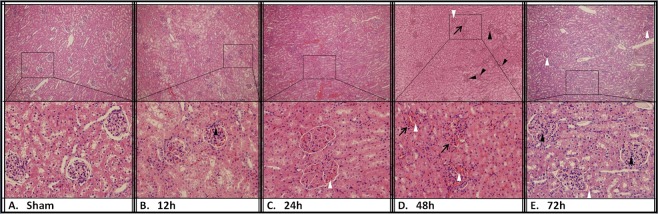
H&E staining of the kidney tissue. 36 rabbits (6 rabbits per group) were subjected to cholecystocolonic anastomosis and kidney tissue samples were harvested. H&E staining of the kidney tissue at 12 h (**C**), 24 h (**D**), 48 h (**E**), and 72 h (**F**) time point after modeling versus sham group (**B**). The white triangles indicate varying degrees of congestion, the black arrows indicate damage of basement membranes, and the black triangles indicate inflammatory infiltration in glomerulus. (Original magnification: × 100 and × 400).

**Figure 7:**
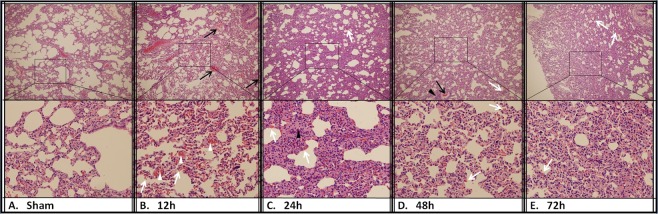
H&E staining of lung tissue. 36 rabbits (6 rabbits per group) were subjected to cholecystocolonic anastomosis and lung tissue samples were harvested. H&E staining of the lung tissue at 12 h (**C**), 24 h (**D**), 48 h (**E**), and 72 h (**F**) time point after modeling versus sham group (**B**). The white triangles indicate congestion of pulmonary mesenchyme, the white arrows indicate hemorrhage of alveolae, and the black arrows indicate obvious inflammatory infiltration. (Original magnification: × 100 and × 400).

The results of histologic examination were presented in Figs 5–7. The liver of CCA rabbits showed characteristic changes of reflux cholangitis (Fig. 5B–F). Extensive edema with mild congestion in hepatic sinusoid and inflammatory cell infiltration in some portal areas were visible at 12 h after CCA. Hydropic degeneration of hepatocytes and structural disorder of hepatic lobules with large vesicular structures occurred at 24 h after the operation. There was visible interstitial fibrosis, evident inflammatory infiltration, suppuration in the bile duct, and cholangiectasis with hyperplasia of small bile ducts in the portal area (Fig. 5D). Whereafter, there was infiltration of macrophages and fusion foreign-body giant cells with bacteria in the portal area, where patchy necrosis of nearby hepatocytes was observed and aggravated from the periphery to the hepatic centrilobular portion at 48 h after the operation (Fig. 5E). At 72 h after CCA, there were severe fibrosis and hyperplasia of small bile ducts in the portal area, accompanied by purulent focus with aggregate inflammatory cells, large flakes showing coagulative necrosis and progressive necrosis with hyperchromatism at its edge (Fig. 5F). Substantial kidney injury was also presented after CCA. There was significant edema and ballooning degeneration of renal tubular epithelial cells, mild inflammatory infiltration in renal corpuscles at 12 h after CCA (Fig. 6B). At 24 h after CCA, mild inflammatory infiltration with occasionally incomplete basement membranes in the glomerulus, renal tubular injury with interstitial congestion were observed (Fig. 6C). Glomeruli congestion and increased inflammatory infiltration were accompanied by extensive interstitial congestion at 48 h after CCA (Fig. 6D). Congestion continued at 72 h post CCA (Fig. 6E). Significant changes in lung pathomorphology were observed at 12 h after CCA. Congestion and edema of pulmonary mesenchyme and pulmonary alveoli caused by the enhancement of pulmonary alveolus-capillary membrane permeability, internal hemorrhage of bronchioli terminales were observed (Fig. 7B). There was evidence of interstitial pneumonia with aggravating inflammatory cell infiltration, hemorrhage and hyperplasia of alveolar epithelium at 24 h post CCA (Fig. 7C). The interstitial pneumonia was persisted with inflammatory cell infiltration at 48 h (Fig. 7D) and 72 h after CCA (Fig. 7E).

## Discussion

Animal models are essential for modern medical research. In the current study, we characterized the pathophysiologic changes of a novel rabbit model of BTI-derived sepsis. We found CCA in rabbits mimicked many features of clinical BTI and could be used as a suitable model for BTI or sepsis research.

Bacteremic BTI is life-threatening. Patient’s condition can deteriorate rapidly due to the development of sepsis^18^. Bacterial culture is a very important technique for early diagnosis, disease evaluation, and anti-infective treatment. Organisms cultured from the bile of patients with acute cholangitis have been found to be predominantly polymicrobial, including *Escherichia coli*, *Enterococcus*, and *Enterobacter*. The most common anaerobic organism is Clostridium spp^19,20^. Previous observations show that about 90% of bile cultures were positive. Blood cultures are found to be positive in 21%–71% of patients, with only one type of organism isolate^21–23^. The results of bacterial culture in our model were a very strong indication of the polymicrobial nature of acute BTI. The CCA model not only shows similar strains of aerobic and anaerobic organisms in culture and a positive rate in both liver and blood but also mimics the growth trend of bacteria in severe cholangitis. This makes this model a good tool for testing antibiotic treatments.

White-cell and platelet counts in blood tests are routinely used for diagnosing and assessing severity of BTI^24^. The hematological changes in the CCA model are consistent with BTI-derived sepsis. The red-cell count and hemoglobin levels were not affected by the surgery or infection. White-cell and neutrophil granulocyte counts tended to increase, while the platelet count tended to decrease after CCA. Sepsis caused by acute hepatobiliary infection also initially affects liver function. Jaundice, which is one of three components of the Charcot triad, is commonly used for clinical diagnosis of acute cholangitis. Hyperbilirubinemia is one of the diagnostic criteria for severe sepsis or septic shock^25^. In our model, dissociation of bilirubin and liver enzymes are consistent with the condition of advanced liver dysfunction. Albumin levels also decreased along with injection. Transient renal dysfunction was also observed in our model.

Sepsis is usually associated with an exacerbated inflammatory response^26^. Dysregulated expression of the cytokines TNF-α and IL-6 has been found to correlate with sepsis mortality^27,28^. Previous studies have also implicated IL-10 as an important regulator of septic shock^29^. These plasma cytokine levels were found to be markedly increased at the first 12 h in the present study, a period representing the early inflammatory phase of sepsis. HMGB1 was considered to be a late-phase inflammatory mediator that functions as a damage-associated molecular pattern^30^. It was found to be released during the late stage of sepsis by activated immune cells and necrotic tissue^31^. However, in the current study, HMGB1 increased quickly during the early phase of sepsis, which is 12 h after the model was established and then dropped rapidly, a similar pattern as TNF-α. This result is consistent with what was reported in patients with acute obstructive suppurative cholangitis-induced sepsis^32^, suggesting HMGB1 can also increase early after sepsis.

Acute organ dysfunction of the cardiovascular system is often visible in sepsis and leads to hemodynamic changes^33^. Hypotension is one of the defining characteristics of septic shock, but humans with clinical sepsis could develop both hyperdynamic and hypodynamic shock. Most animal models of sepsis only produce the hypodynamic response with reduced cardiac output and hypotension, which is inconsistent with human sepsis^34^. Our model has the advantage of producing increased cardiac output and heart rate and reduced systemic vascular resistance during the first 12 h. Then systemic hypotension gradually emerged. This might make the CCA model well suited for testing resuscitative therapies. Larger animals also make for easier study of hemodynamics, especially changes in blood flow and echocardiography.

Severe sepsis and septic shock can cause life-threatening organ dysfunction through a dysregulated host response to infection^35^. The liver is a target for sepsis-related injury. The liver is essential to the regulation of immune defense and plays a central role during sepsis^36^. The CCA model was found to mimic the characteristic pathological findings of acute liver injury with hepatogenous infection. Acute organ dysfunction most commonly affects the respiratory system and is classically manifested as acute respiratory distress syndrome (ARDS), and the kidneys are also often affected^33,37^. The characteristic pathological findings in the acute phase of clinical ARDS were interstitial and alveolar edema with accumulation of inflammatory cells^38^. For acute kidney injury, early studies examined renal biopsies from patients with septic shock, showing differing degrees of acute tubular lesions along with infiltration of leucocytes^39^. In the present study, the CCA model was found to simulate similar pathological manifestations.

Sepsis is a complex syndrome associated with a series of clinical features and inflammatory responses^40^. The pathological manifestations of sepsis include infection, hemodynamic variables, elevated plasma inflammatory factors, and organ dysfunction. A suitable animal model should essentially mimic the pathophysiological mechanisms of sepsis in humans^9^. Various investigators have utilized the model of cecal ligation and puncture (CLP) to produce polymicrobial sepsis in rodents^41,42^. The rodent model of CLP is considered to be clinically relevant since it mimics many features of clinical peritonitis-sepsis. Therefore, the CLP model of sepsis remains the gold standard for sepsis research. The CLP model of sepsis has also been exploited in rabbits^43,44^. The great lethality and polymicrobial origin of infections make it an excellent model to test the efficacy of experimental treatment of sepsis. However, the hemodynamic feature of this model has not been fully investigated. Also, the pathologic changes in clinical sepsis are highly variable due to differences in the initial site of infection, the causative organism, and the organs involved. the pathologic changes in clinical sepsis are highly variable due to differences in the initial site of infection, the causative organism, and the organs involved^45^. In this study, cholecystocolonic anastomosis was established to simulate polymicrobial sepsis caused by acute BTI, such as cholangitis and cholecystitis. Contents of the colon with multiple organisms entered the hepatobiliary system, and bacteria were slowly released into the blood, causing sepsis. The median survival time of this model was 96 h, which provides an extended therapeutic and research window. We believe our CCA model is a good supplement to the CLP sepsis model.

## Conclusions

In summary, cholecystocolonic anastomosis produces polymicrobial sepsis in rabbits, which mimics many aspects of human BTI-derived sepsis. It is reproducible and easy to perform and may serve as an excellent model for future sepsis research.

## Data Availability

All datasets used and/or analyzed during the current study are available from the corresponding author upon reasonable request.
